# Arterial spin labeling perfusion in acute Wernicke encephalopathy: a case series discussion

**DOI:** 10.1259/bjrcr.20220137

**Published:** 2023-10-24

**Authors:** Phoebe Ann, Mark Chen, Thomas Naidich, Puneet Belani, Kambiz Nael

**Affiliations:** 1 Department of Radiological Sciences, David Geffen School of Medicine, University of California Los Angeles, 757 Westwood Plaza, Los Angeles, California; 2 Department of Diagnostic, Molecular and Interventional Radiology, Icahn School of Medicine at Mount Sinai, 1176 5th Ave, New York, NY, United States

## Abstract

Wernicke’s encephalopathy (WE) is a life-threatening neurologic disorder resulting from thiamine (vitamin B1) deficiency that can be secondary to chronic alcohol abuse, gastrointestinal surgery, systemic infectious and non-infectious diseases, and chemotherapy. WE is classically characterized on MRI by reduced diffusion and T2 prolongation along the mammillothalamic tracts, periaqueductal gray and tectal plate. We present two patients with acute WE who had baseline arterial spin labeling (ASL) perfusion at the time of presentation, demonstrating increase in cerebral blood flow (CBF) within the classically involved brain regions and concurrent global cerebral cortical hypoperfusion. Both patients were successfully treated with intravenous thiamine infusion. Post-treatment MRI demonstrated improvement of reduced diffusion and normalization of CBF within the involved structures. Prior histopathological studies have documented prominent undulation and luminal dilatation of arteries and arterioles in acute WE lesions, likely explaining the increased perfusion shown by imaging. The root of this pathophysiologic process may trace back to thiamine’s biochemical role in maintaining osmotic gradients and glucose metabolism, that if failed can lead to arterial hyper-perfusion.

Our findings show that ASL-CBF can highlight the underlying pathophysiology in patients with acute WE by demonstrating increased CBF in involved central structures. This luxury perfusion may be a compensatory or protective mechanism by which increased metabolic demand is met in the acute setting and which, if treated timely, will show normalization of CBF on ASL imaging.

## Learning points

- Acute Wernicke’s encephalopathy (WE) manifests on MRI as T2 prolongation and restricted diffusion along several classic regions including the mammillothalamic tracts, periaqueductal gray, and tectal plate.
- Hyperperfusion on arterial spin labeling (ASL) is seen at the classically involved regions in acute WE and may offer insight into the pathophysiology of the disease state.
- Hypoperfusion in the cerebral cortex in acute WE may correspond to clinical encephalopathy.
- The MRI findings of patients who receive prompt treatment of acute WE demonstrate normalization of cerebral blood flow.

## Background

Wernicke’s encephalopathy (WE) is a life-threatening neurologic disorder due to thiamine (vitamin B1) deficiency. The exact prevalence and incidence are unknown but some autopsy studies suggest a prevalence of 0.5–2.8%, with the vast majority of WE patients diagnosed postmortem.^1^ The classical clinical triad of encephalopathy, oculomotor abnormalities, and gait ataxia are only present in 10% of patients.^2^ Most patients exhibit a wide array of nonspecific symptoms, for example encephalopathic behavior ranging from indifference and inattentiveness to loss of consciousness. The variability of symptom presentation makes clinical diagnosis difficult.

**Figure 1.**
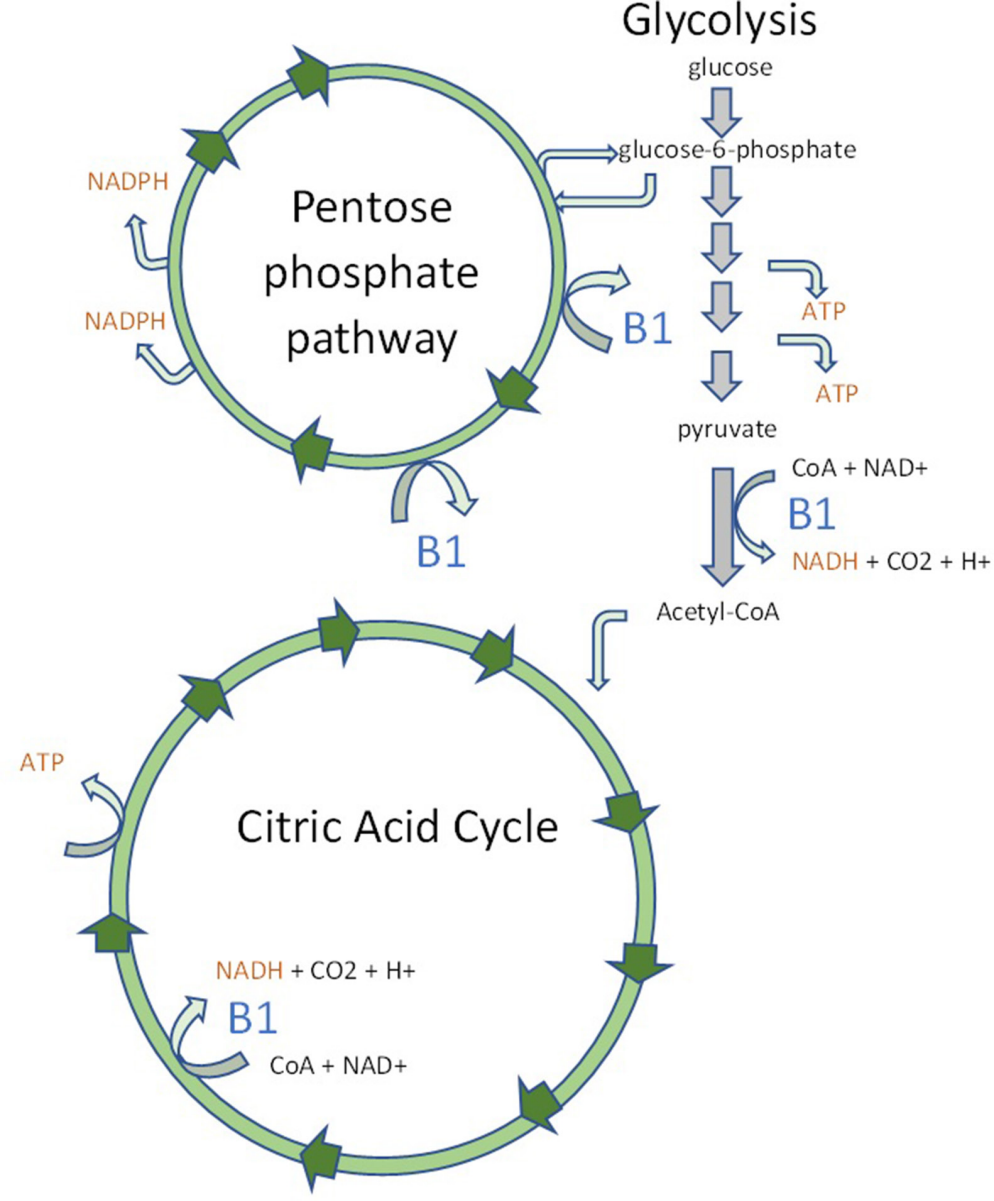
Adapted from Edwards K. A.^3^ Simplified diagrams of the citric acid cycle, glycolysis, and the pentose phosphate pathway. Thiamine or B1 in blue indicates B1-dependent enzymes. The end products nicotinamide adenine dinucleotide NADH, nicotinamide adenine dinucleotide phosphate NADPH, and adenosine triphosphate ATP in orange are highlighted to indicate products of these biochemical pathways key to cell integrity.

In the absence of thiamine (B1), a combination of cytotoxic and vasogenic edema drives the pathophysiology of acute WE. B1 is an essential cofactor for many key metabolic pathways including the citric acid cycle and pentose phosphate pathway (Figure 1).^3^ With B1 deficiency, the citric acid cycle halts, causing depletion of adenosine triphosphate (ATP), and nicotinamide adenine dinucleotide (NADH).^4^ Depletion of ATP results in failure to maintain osmotic gradients dependent on active transport. Depletion of NADH results in inability to neutralize free radicals, hydrogen ions (H+), and intermediate metabolites such as glutamate and lactate. Additionally with halting of the pentose phosphate pathway, there is a decreased DNA/RNA ratio, increasing oxidative stress in the cell. In sum, these metabolic changes cause the cell to undergo cytotoxic edema. Critically, at the blood-brain barrier, the cells responsible for barrier integrity (capillary endothelial cells, pericytes, and astrocytes) are exposed to the increased synaptic glutamate concentrations, leading to excitotoxicity which triggers vasogenic edema in the brain.

Animals, unlike plants, require exogenous B1 intake. Absorption occurs through the jejunal brush border mucosa via B1 transporters including human thiamine transporters hTHTR-1 and hTHTR-2.^5^ These cell membrane transporters are H + and ATP-dependent. Causes of WE include nutritional deficiency or impairment of jejunal mucosal absorption, for example disruption of pH balance from chronic alcohol abuse, bariatric surgery or prolonged vomiting, or from sloughing of high-turnover cells including brush border cells during chemotherapy.

**Figure 2.**
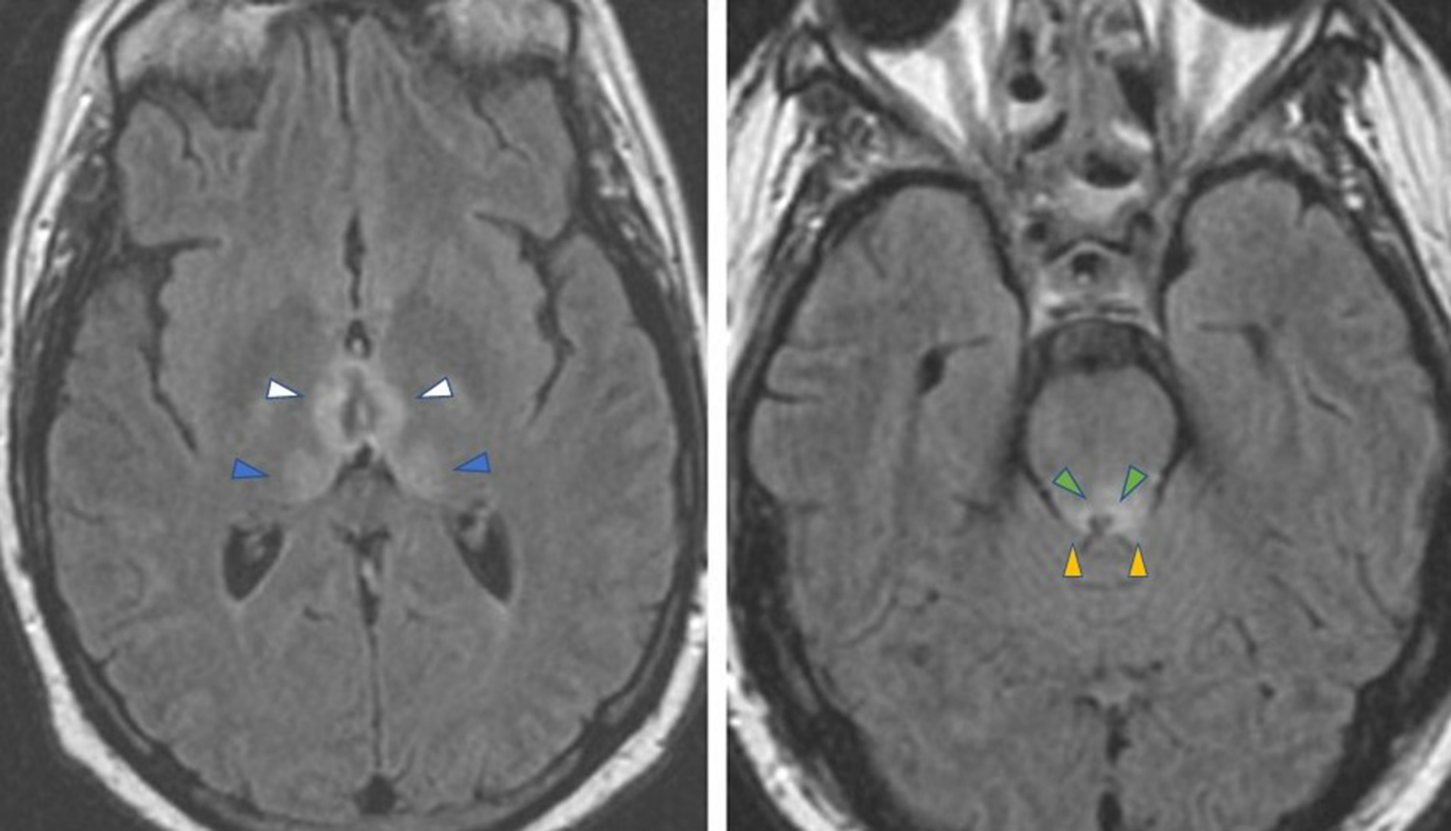
Axial T2-FLAIR images in a patient with acute WE are shown. Typical MRI findings in acute WE include T2 prolongation along the medial thalami (blue arrowhead) and mammillothalamic tracts (white arrowheads) on the left image, as well as the periaqueductal gray (green arrowheads) and tectal plate (yellow arrowheads) on the right image.

The classic MRI findings of WE are well documented and include T2 prolongation with variable degree of reduced diffusion or enhancement involving classically the mamillary bodies, medial thalami, hypothalami, mammillo-thalamic tracts, periaqueductal gray, and tectal plate (Figure 2).^6^ It has been reported that WE selectively affects these central structures because osmotic gradients at these regions are highly dependent on B1. The mainstay of treatment is prompt intravenous thiamine repletion, although this is often delayed by the difficulty of clinical diagnosis. If left untreated, mortality can be as high as 20%.

We present two patients with acute WE who had baseline arterial spin labeling (ASL) perfusion studies at the time of presentation, demonstrating (i) increase in cerebral blood flow (CBF) within the characteristically involved central brain regions and (ii) global cerebral cortical hypoperfusion. Post-treatment MRI demonstrated significant improvement of imaging findings including improvement in conventional imaging findings and also correction of CBF in WE lesions and cerebral cortices.

MR image acquisition was performed using a 1.5 T MR scanner (GE Optima MR450w; GE Medical Systems, Milwaukee). ASL was performed using a 3D pseudocontinuous ASL (pCASL) with a fast spin echo stack-of-spiral readout with eight interleaves and the following parameters: TR/TE 4525/11 ms, FOV: 240 mm^2^, matrix size: 64 mm^2^, 30 slices each 4 mm thick, and NEX = 3. Each spiral arm included 512 sampling points. Pseudocontinuous spin labeling was performed for 1.5 sec before a post–spin-labeling delay of 2 s. This setting resulted in acquisition of 3D voxel size of 3.8 × 3.8×4 mm^3^ during 4:30 min. Image reconstruction was performed online by the scanner software. Pairwise subtraction between label and control images was obtained and averaged to generate the mean difference and converted to CBF maps.

## Case Report 1

**Figure 3A.**
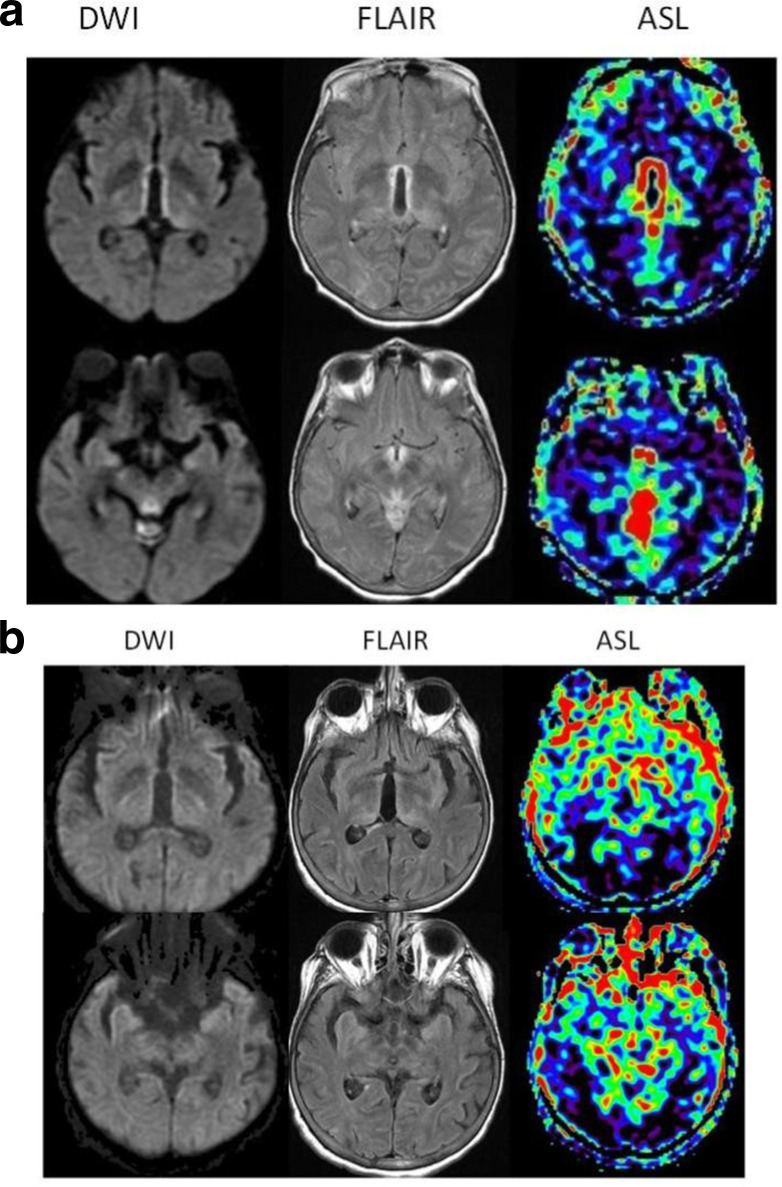
(a) Pre-treatment MRI of Patient 1 demonstrates restricted diffusion and T2 prolongation along the classically involved regions of the medial thalami, mammillothalamic tracts, mammillary bodies, and periaquectal gray. These lesions correspond to increased CBF on ASL, with diffuse cerebral cortical hypoperfusion. (b): Post-treatment MRI of Patient 1 demonstrates improvement of restricted diffusion and T2 prolongation along the medial thalami, mammillothalamic tracts, mammillary bodies, and periaquectal gray. There is corresponding normalization of CBF in these regions, as well as improvement of previously seen cortical hypoperfusion.

A female in her 70 s, with a previous medical history of end-stage renal disease status post kidney transplant, presented with several days of acute-onset confusion, weakness, and urinary incontinence. She was found to be septic due to vancomycin-resistant enterococcus urinary tract infection (UTI) and was started on antibiotics. Despite several days of antibiotic treatment, she exhibited worsening delirium and lethargy. A lumbar puncture was performed which was unremarkable. However, her serum B1 was 35 (normal range 74–222 nmol l^−1^). Her MRI Brain showed T2 prolongation along with restricted diffusion and increased ASL-CBF involving the medial thalami, mammillothalamic tracts, and periaqueductal gray. ASL also demonstrated global cerebral cortical hypoperfusion. With these classic MRI findings and serum biochemical confirmation, she was diagnosed with acute WE and subsequently treated with five days of IV thiamine repletion. Her post-treatment MRI demonstrated improvement in T2 prolongation and normalization of reduced diffusion. There was also correction of CBF involving the WE lesions and normalization of global cortical hypoperfusion. After gradual improvement of mental status, placement of tracheostomy and jejunal tube, and completion of IV antibiotics with clinical defervescence, the patient was stable enough for transfer to long term acute care facility. Figure 3A and B show the imaging findings in this patient before and after treatment respectively.

## Case Report 2

**Figure 4A.**
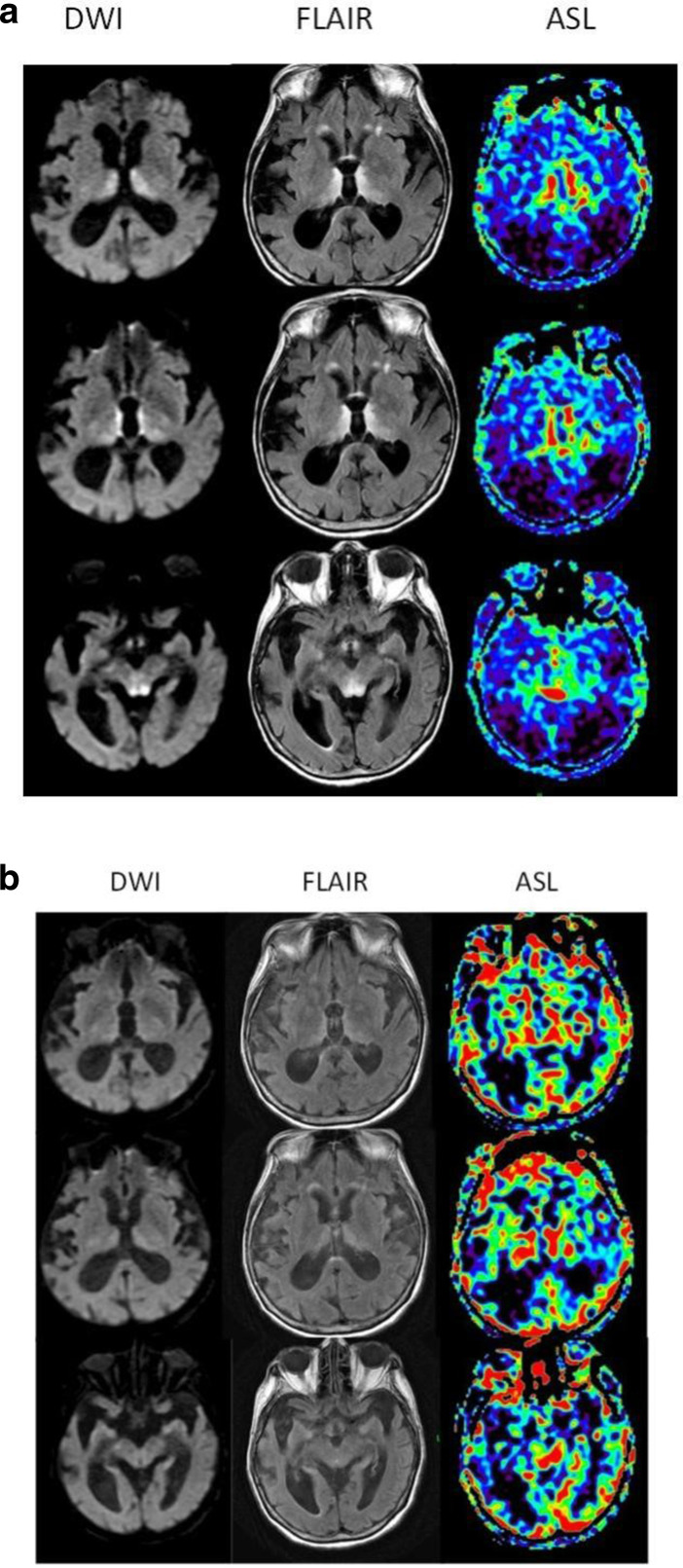
(a) Pre-treatment MRI in Patient 2 demonstrates restricted diffusion and T2 prolongation in the medial thalami, mammillothalamic tracts, and periaqueductal gray. These areas again corresponded to increased CBF on ASL, with relative cerebral cortical hypoperfusion. (b) Post-treatment MRI in Patient 2 showed improvement of restricted diffusion and T2 prolongation of the medial thalami, mammillothalamic tracts, and periaqueductal gray, along with normalization of the increased CBF previously seen on ASL. There is correction of prior global cerebral hypoperfusion.

A female in her 70 s with a previous medical history of major depressive disorder with psychosis presented with acute paranoia. Her current psychiatric medications were up-titrated with plans to start electroconvulsive therapy. However, several days into her hospitalization she became acutely obtunded with sluggish pupils and flaccid paresis. A lumbar puncture was performed which was unremarkable. EEG was negative for seizure activity. However, her plasma B1 was found to be low at 26 nmol l^−1^ (normal 74–222 nmol l^−1^). MRI showed T2 prolongation with restricted diffusion and increased ASL-CBF involving the medial thalami, mammillothalamic tracts, periaqueductal gray, and tectal plate. There was also cortical hypoperfusion with posterior predilection. Thus, she was diagnosed with WE and subsequently started on IV thiamine repletion. Her post-treatment MRI demonstrated improved T2 prolongation, resolution of restricted diffusion and correction of CBF within the central WE lesions and normalization of cortical hypoperfusion. She demonstrated limited improvement in mental status. Her hospital course was complicated by aspiration pneumonitis and sacral decubitus ulcer. She was ultimately stabilized to undergo tracheostomy and PEG placement, after which she was successfully transferred to skilled nursing facility. Figure 4A and B show the imaging findings in this patient before and after treatment, respectively.

## Discussion

Our findings show that in patients with acute WE, addition of ASL to the standard diagnostic brain MRI can display elevation of CBF in the classically involved central brain regions in addition to cerebral cortical hypoperfusion. Further, these findings are reversible after timely B1 repletion. Our results are in concordance with recently published results showing the ability of ASL to detect hyperperfusion in the classically involved WE central brain structures.^7^

The hyperperfusion seen on MRI in acute WE is explained in part by prior animal and postmortem histopathological studies. Earlier experimental mice models have shown vascular hyperpermeability and hemorrhage in regions affected by WE. Furthermore, the vascular permeability was reversed in mice who responded to B1 repletion.^8–10^ In addition, postmortem histopathological studies in humans have shown luminal dilatation of arteries, endothelial swelling, and intramural and perivascular fibrin exudation and petechial hemorrhage in patients with WE. These features were identified exclusively in the arterioles and arterial side-of the capillaries.^11^ In another study comparing MRI and autopsy findings, the characteristic T2 prolongation noted in the periaqueductal gray, and around third and fourth ventricles correlated with spongy disintegration of neuropil with neuron sparing, swelling of capillary endothelial cells, and extravasation of red blood cells.^12^ Such reported arterial dilatation and increased arterial permeability helps to explain initial hyperperfusion seen in the early stages of WE.

The increased CBF found in acute WE lesions may also reflect a compensatory mechanism to deliver more substrates (oxygen and glucose) to affected regions depleted of energy. As discussed earlier B1 deficiency can result in ATP depletion and insufficient energy resulting in restricted diffusion on MRI.^3^ There is a well-established correlation between depleted ATP reserve and restricted diffusion.^13^ Early experimental animal models demonstrated restricted diffusion on MRI in rat cortex after administration of a selective inhibitor of the sodium (Na)/ potassium (K) ATP-ase pump.^14^ Similarly, after cerebral ischemia was induced by middle cerebral artery occlusion, Na/K ATP-ase activity and intracellular K concentration were decreased, while intracellular Na concentration and water content increased in the portions of the ischemic cortex which demonstrated restricted diffusion on MRI.^15^ As local cerebral blood flow drops below a critical threshold (15–20 mL/ 100 g / min), the energy supply is not sufficient to sustain the Na/K ATP-ase pump, causing an acute influx of water to the intracellular space, which then manifests as DWI hyperintensity on MRI.^16^

It is possible that in the presence of an energy-demand imbalance, a compensatory increase in cerebral perfusion can serve to sustain demand until correction is made. This diffusion-perfusion coupling has been reported in similar processes such as seizure where there is an acute energy-demand imbalance. For example, several studies have shown that epileptogenic foci represented by cortical FLAIR and DWI hyperintensities in status epilepticus patients demonstrate peri-ictal hyperperfusion followed by post-ictal hypoperfusion.^17^ Given the well-established phenomenon that prolonged ictal activity increases tissue glucose metabolism, the ictal hyperperfusion may serve as a compensatory response to attempt to meet energy demand.^18,19^ Alternatively, hyperperfusion may result from a loss of autoregulation in cerebral blood flow in anoxic states, due to the loss of cerebral arterial tone, and due to the buildup of metabolites such as carbon dioxide, adenosine, potassium, and nitric oxide.^20^ Notably, carbon dioxide is a potent vasodilator and may explain the hyperperfusion on ASL seen in patients with hypercapnia and hypoxic ischemic encephalopathy.^21^

Another important finding in acute WE is cerebral cortical hypoperfusion, which was depicted on ASL in our patients. Cortical hypoperfusion and decrease in cerebral CBF in patients with WE has been demonstrated by other imaging modalities such as Xenon-133 CT,^22^ or positron emission tomography (PET) labeled oxygen-15.^23^ Cerebral cortical hypoperfusion is very likely an imaging manifestation of clinically identified encephalopathy. It goes hand-in-hand with the pathophysiology of thiamine deficiency, in that dysfunction in maintaining osmotic gradients and glucose metabolism, and specifically disturbing neurotransmitter function in the brain, may lead to hypometabolism and hypoperfusion. Furthermore, global cortical hypoperfusion on ASL has been identified in other clinically similar states of encephalopathy, including the post-ictal period,^24^ and in both animal and human studies on neurodegenerative disease such as Alzheimer’s and CADASIL.^25^

In summary, ASL can highlight the underlying pathophysiology in patients with acute WE by demonstrating increased CBF in involved central structures. This luxury perfusion is likely due to arterial dilatation and increased arterial permeability in the affected regions. It is likely a compensatory or protective mechanism by which increased metabolic demand is met in the acute setting. In addition, ASL can depict the cerebral cortex hypoperfusion that may be responsible for altered mental status and encephalopathy in patients with acute WE. Our findings suggest that as ASL is becoming mainstream and broadly available, addition of ASL (about 4 min acquisition) to routine brain MRI may provide additional physiological information in patients with suspected acute WE similar to those obtained previously by Xenon CT or PET.
